# Cross-approximate entropy of cortical local field potentials quantifies effects of anesthesia - a pilot study in rats

**DOI:** 10.1186/1471-2202-11-122

**Published:** 2010-09-23

**Authors:** Matthias Kreuzer, Harald Hentschke, Bernd Antkowiak, Cornelius Schwarz, Eberhard F Kochs, Gerhard Schneider

**Affiliations:** 1 Department of Anesthesiology, Klinikum rechts der Isar, Technische Universität München, München, Germany; 2 Section of Experimental Anaesthesiology, University Hospital Tübingen, Tübingen, Germany; 3 Department of Cognitive Neurology, Hertie-Institute for Clinical Brain Research, University Tübingen, Tübingen, Germany; 4 Werner Reichardt Centre for Integrative Neuroscience, University Tübingen, Tübingen, Germany

## Abstract

**Background:**

Anesthetics dose-dependently shift electroencephalographic (EEG) activity towards high-amplitude, slow rhythms, indicative of a synchronization of neuronal activity in thalamocortical networks. Additionally, they uncouple brain areas in higher (gamma) frequency ranges possibly underlying conscious perception. It is currently thought that both effects may impair brain function by impeding proper information exchange between cortical areas. But what happens at the local network level? Local networks with strong excitatory interconnections may be more resilient towards global changes in brain rhythms, but depend heavily on locally projecting, inhibitory interneurons. As anesthetics bias cortical networks towards inhibition, we hypothesized that they may cause excessive synchrony and compromise information processing already on a small spatial scale. Using a recently introduced measure of signal independence, cross-approximate entropy (XApEn), we investigated to what degree anesthetics synchronized local cortical network activity. We recorded local field potentials (LFP) from the somatosensory cortex of three rats chronically implanted with multielectrode arrays and compared activity patterns under control (awake state) with those at increasing concentrations of isoflurane, enflurane and halothane.

**Results:**

Cortical LFP signals were more synchronous, as expressed by XApEn, in the presence of anesthetics. Specifically, XApEn was a monotonously declining function of anesthetic concentration. Isoflurane and enflurane were indistinguishable; at a concentration of 1 MAC (the minimum alveolar concentration required to suppress movement in response to noxious stimuli in 50% of subjects) both volatile agents reduced XApEn by about 70%, whereas halothane was less potent (50% reduction).

**Conclusions:**

The results suggest that anesthetics strongly diminish the independence of operation of local cortical neuronal populations, and that the quantification of these effects in terms of XApEn has a similar discriminatory power as changes of spontaneous action potential rates. Thus, XApEn of field potentials recorded from local cortical networks provides valuable information on the anesthetic state of the brain.

## Background

It has long been known that anesthetics alter neuronal activity of the brain, yet it is still unclear which of the alterations of brain activity are instrumental in causing sedation, amnesia and unconsciousness. Anesthetics likely disrupt communication between cortical areas [1], and thus impair large-scale integration of information hypothesized to be a prerequisite to proper brain function, particularly conscious perception [2-5]. A number of experimental observations are in agreement with this concept. In rats stimulated with light flashes, volatile anesthetics disrupted anterior-posterior phase synchronization of field responses [6] and depressed long-latency spike responses in visual cortex, thought to arise from cortico-cortical interactions [7]. In humans, during the transition from waking to loss of consciousness, various general anesthetics decoupled gamma rhythms between anterior and posterior cortical areas as well as between homologous areas in different hemispheres [8]. Employing transcranial magnetic stimulation, Ferrarelli et al. recently demonstrated a breakdown of cortical effective connectivity upon loss of consciousness induced by midazolam [9].

A complementary insight into network effects of anesthetics was recently provided by Hudetz and colleagues, who demonstrated that in rats volatile anesthetics diminished the independence of spontaneous electroencephalographic (EEG) signals recorded from different hemispheres [10]: isoflurane, and halothane above 0.4%, reduced cross-approximate entropy (XApEn), a nonlinear information statistical parameter which quantifies the independence (or dissimilarity) of signals. Fittingly, cholinergic stimulation reversed the isoflurane-induced decrease of XApEn [11]. These findings suggest that anesthetics induce changes of thalamocortical networks and slow predominant frequencies. This impairs brain function by promoting uniformity of signals and impeding an exchange of independent information between cortical areas, as a contrast to the transient synchronization of fast oscillations which has been suggested as the mechanism underlying conscious perception [2].

It is less clear at present in which manner anesthetics impair information processing on a much smaller spatial scale. In local cortical networks, subnetworks defined by strong excitatory connections exist which may operate quite independently of each other [12,13]. With anesthetics, their independence of operation may be compromised by a general decrease of cortical neuronal excitability [14-18]. Furthermore, inhibitory interneurons project locally and have a great potential to pace their postsynaptic targets [19,20]. Under conditions of pharmacologically enhanced GABAergic transmission, they may coerce independent subnetworks into more synchronous, uniform activity patterns [21,22].

In the present pilot study, we investigated to what degree anesthetics alter signal independence in the somatosensory ('barrel') cortex of the rat. We recorded spontaneous local field potential (LFP) activity from multiple, closely spaced electrodes, which sample from a much smaller subset of neurons than EEG electrodes [23,24]. The effects of the volatile anesthetics isoflurane, enflurane and halothane were evaluated in three animals. Our results show that the independence of activity patterns across recording sites as quantified by XApEn is a monotonously declining function of anesthetic concentration, suggesting that volatile anesthetics strongly promote uniform activity already on the level of local neocortical circuits.

## Results

**Figure 1:**
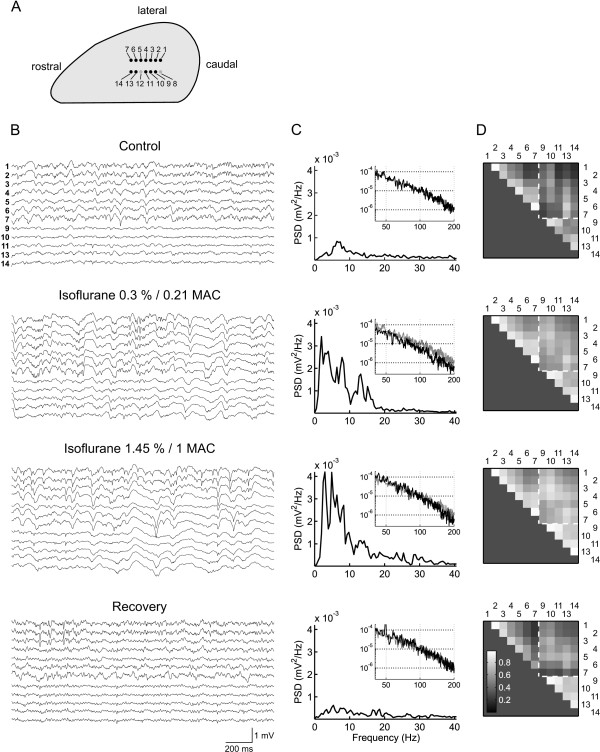
**Signal characteristics of neocortical local field potentials**. **A**, top view (schematic) of a rat cortical hemisphere and approximate location of the recording electrode array in one animal (rat1). The electrodes were arranged in two rows and placed in the primary somatosensory (barrel) cortex, parallel to the rostrocaudal axis (sizes are not to scale). Gray dots correspond to dysfunctional electrodes. Due to the curvature of the neocortex electrode row 2 was in a deeper cortical lamina as compared to row 1. **B**, exemplary raw local field potential excerpts of 2 s length, filtered between 0.5 and 200 Hz, from the same animal at control, at a sedating concentration and an anesthetic concentration of isoflurane and after recovery. Note the similarity of the signals within each row (electrodes 1-7 and 9-14) and particularly between adjacent pairs of electrodes. **C**, power spectral density (PSD) plots of all raw data segments selected for analysis (channel 1 only). The insets depict the PSD in the range 40-200 Hz in double logarithmic coordinates. For comparison, the PSD under control conditions is replotted in gray in the background. **D**, Cross correlation of the same data as depicted in C. Each grayscale-coded matrix contains values obtained from non-redundant pairwise combinations of recording sites (except auto-combinations) as shown in A. Channel numbers are given at the top and to the right of each matrix. The white broken lines separate within-row cross correlations (upper and lower sub-triangles) and between-rows cross correlations (rectangular area). Note that within-row cross correlations decrease in a monotonic fashion with distance between the electrodes in a pair.

Figure 1 illustrates the location of the electrode arrays within neocortex and shows exemplary recordings from an experiment in which isoflurane was administered. Under control conditions, spectral power of the LFP peaked at 6-7 Hz and gradually declined towards higher frequencies (Figure 1C, top). The signals recorded from different electrodes seemed similar to varying degrees. In particular, similarity between signals depended on the spatial arrangement of the electrodes: within each row, adjacent electrodes recorded signals which were in general more similar to each other than electrodes placed further apart. This is evident in the pattern of cross correlation values in Figure 1D (top). Moreover, the signals from electrode row 1 had a higher amplitude than those from row 2 and qualitatively appeared somewhat different. The qualitative difference in signal appearance between rows was reflected in cross correlation values which were on average lower than within-row correlations (Figure 1D, top; compare upper right rectangular area with the two triangular areas, separated by white broken lines). We attribute the differences in signal amplitude and appearance between electrode rows to the different layers in which they were located (see Methods; [25,26]) as well as to their separation by more than twice the interelectrode distance (500 versus 180 um).

At all concentrations applied, isoflurane greatly enhanced the amplitude of low-frequency signal components (up to ~20 Hz) and reduced high frequency components (above 100 Hz, Figure 1B, C). At the highest concentration (1.45%), high-amplitude LFP 'spikes' occurred, and in one animal burst suppression patterns appeared. Enflurane and halothane led to signal changes which were similar at low concentrations, but at concentrations close to 1 MAC the anesthetics clearly differed in their tendency to produce high-amplitude LFP spikes and burst suppression patterns (enflurane > isoflurane > halothane). Both the shifts in the spectral composition of neural activity common to all three anesthetics and the agent-specific differences are in agreement with electroencephalographic observations in rats [10,11,18,27-29].

**Figure 2:**
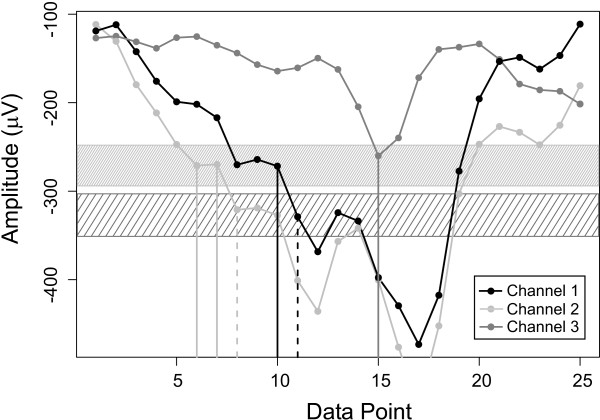
**Calculation of XapEn**. Short excerpt of raw data from three channels shown to illustrate the computation of XApEn. Parameters were set to r = 23 μV (tolerance) and m = 1 (sequence length). Thus, the 'sequence' length is one data point. For illustration purposes we choose data point i = 10 of Channel 1. Similar sequences (data points of similar amplitude) of Channels 2 and 3 are found within the upper rectangle representing tolerance r, i.e. data points 6 and 7 of Channel 2 and data point 15 of Channel 3. This leads to coefficients $C_{10}^{1}(r)(Channel1||Channel2)=\frac{2}{25-1+1}=\frac{2}{25}$ and $C_{10}^{1}(r)(Channel1||Channel3)=\frac{1}{25}$. In the second pass, sequence length is extended to m+1 = 2 points so that now the two point-sequence Channel 1 (10,11) is under consideration. For all previously identified data points in channels 2 and 3 (namely, those similar to Channel 1 (10)) the algorithm checks if the consecutive data point is similar to Channel 1 (11) and hence a pattern similar to Channel 1 (10, 11) exists. Graphically this means that two consecutive data points of channels 2 and 3 have to reside within the upper and lower rectangle, respectively. Only Channel 2 (7, 8) fulfils this requirement. This leads to coefficients $C_{10}^{1+1}(r)(Channel1||Channel2)=\frac{1}{25}$ and $C_{10}^{1+1}(r)(Channel1||Channel3)=\frac{0}{25}$. This pattern matching procedure is performed for all possible sequences so that: $$\begin{array}{l} XApEn(Channel1||Channel2)=\\ \left(n-m+1)-1\sum_{i}\ln C_{i}^{m}(Channel1||Channel2)-\ln C_{i}^{m+1}(Channel1||Channel2\right) \end{array}$$ If a specific pattern in one channel can not be detected in the other, as in the example above, the corresponding $\ln C_{i}^{m}(x||y)$is undefined. In the analyzed data sets and with the parameters chosen, this case did not occur.

**Figure 3:**
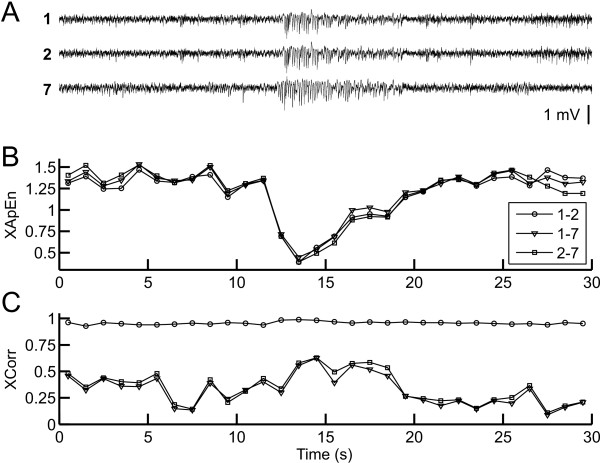
**XApEn compared to cross correlation**. **A**, raw data excerpts of 30 s length from channels 1,2 and 7 of the same recording as shown in Figure 1 (control condition). Note the sequence of 'high voltage rhythmic spikes' (HVRS) starting at ~12 s. **B**, XApEn values calculated from non-overlapping 1 s sequences of all combinations of the three channels shown. **C**, peak cross correlation values of the same channel combinations and time intervals as shown in B. The peak of each non-overlapping 1 s-segment was determined from the respective cross correlation function in an interval of ± 50 ms relative to zero lag.

XApEn quantifies the regularity of patterns in a pair of time series (Figure 2). Before embarking on the time-consuming computation of the entire data set, we were interested in finding out whether it related, qualitatively, to cross correlation, a classical measure of the similarity of signals (see Figure 1D). Figure 3A shows LFP excerpts recorded from three electrodes under control conditions. In contrast to all other data analyzed and presented in this study, this excerpt was chosen to include an episode of 'high voltage rhythmic spikes' (HVRS, see Methods). XApEn values of all three channel combinations (Figure 3B) were very similar throughout the length of the segment although their cross correlations (Figure 3C) differed substantially, in agreement with physical electrode distance. Moreover, XApEn reacted very sensitively to HVRS, declining during this episode. Cross correlation values also changed during HVRS, but the relative change was much smaller than that of XApEn. Thus, this analysis illustrates that XApEn captures characteristics of cortical LFP activity beyond similarity as quantified by cross correlation, and emphasizes that XApEn is well suited to detect changes in cortical network patterns.

**Figure 4:**
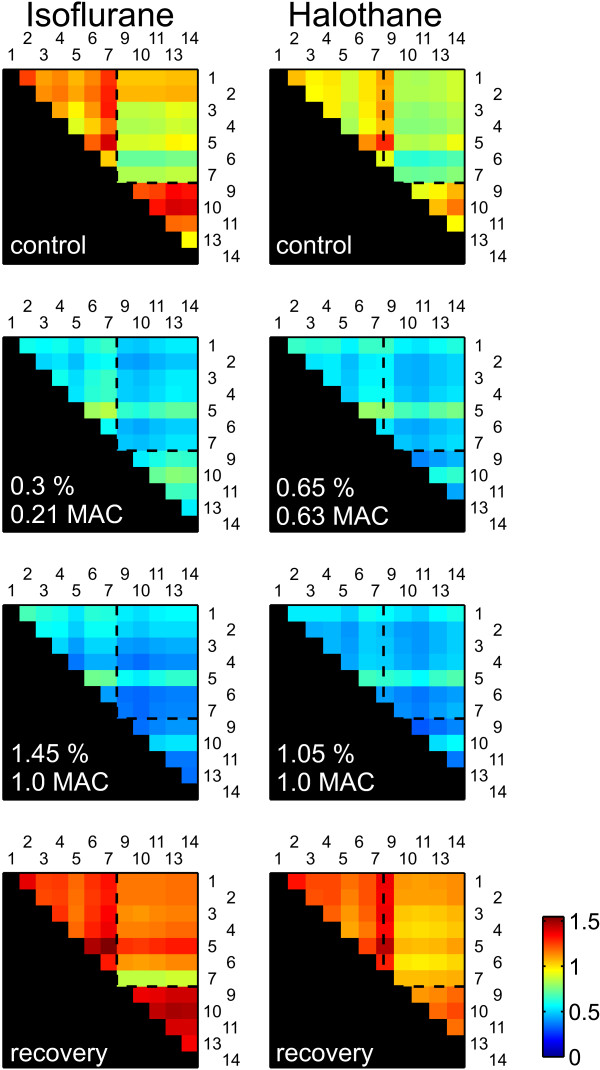
**Anesthetic-induced changes of XapEn**. Each color-coded matrix contains values obtained from non-redundant pairwise combinations of recording sites (cf. Figure 1D). The animal was exposed, in separate experimental sessions, to increasing concentrations of isoflurane (left subcolum) and halothane (right subcolumn). Data are from the same animal as shown in Figure 1.

XApEn values were subsequently computed for all channel combinations and depicted in color-coded matrices. Figure 4 compares two different experimental sessions with one animal, separated by one day, with isoflurane and halothane administration, respectively. As seen under control conditions, the matrices formed fingerprint-like patterns which did not seem to depend on the spatial relations between electrodes. Although the baseline values of XApEn differed, the patterns were quite similar in the two control measurements shown, indicating that no major changes of the cortical network had occurred in the interim. In general, XApEn patterns were remarkably stable over the period of several days in which the experiments were conducted: cross correlations of the linearized XApEn values of the control conditions ranged from 0.86 to 0.99 (median values 0.94, 0.99 and 0.99 for the three animals, each of which was subjected to three recording sessions on separate days).

**Figure 5:**
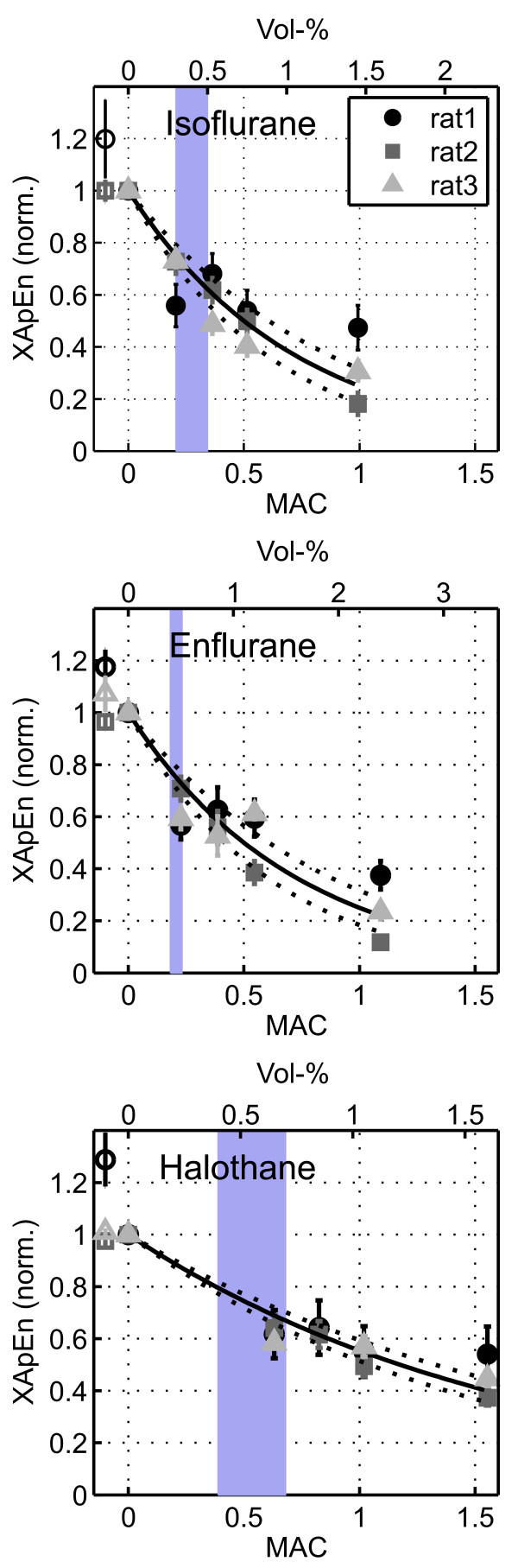
**Dose-response curves of XapEn**. Dependence of cross-approximate entropy on anesthetic concentration expressed in MAC. The data points represent values from all non-redundant pairwise combinations of recording sites, normalized to control and averaged (± standard deviation). Single detached data points at negative abscissa values represent values after recovery (open symbols; no recovery data were recorded for rat 3). The solid lines are fits of the data to the monexponential function y = exp(-x/tau) with tau as the only free parameter. Coefficient of determination (R^2^) values ranged from 0.85 to 0.91, indicating that the fits were adequate. Dotted lines represent upper and lower 95% prediction bounds of the fits. The blue rectangles in the background depict a range of published MAC_awake _values (see main text).

Volatile anesthetics strongly and reversibly decreased XApEn. In the experiments shown in Figure 4, the lowest doses of isoflurane (0.3%) and halothane (0.65%) reduced XApEn values to approximately 50%. Concentration increases to 1 MAC produced only minor additional changes. This was confirmed in dose-response relationships shown in Figure 5. The data points, normalized to control values, were fitted with the monoexponential function y = exp(-x/tau). The free parameter tau, describing the decrease of XApEn with anesthetic concentration expressed in MAC, was as follows [95% confidence intervals]: 0.72 [0.58 0.86] for isoflurane, 0.72 [0.58 0.86] for enflurane and 1.70 [1.49 1.90] for halothane. Thus, halothane affected the cortical network less strongly than isoflurane and halothane. However, all three anesthetics were about equally potent at concentrations corresponding to human MAC_awake_, which render 50% of a human test population unconscious [30-34] and which should be at the lower end of concentrations affecting cortical function of rats (blue rectangles in Figure 5). Both observations are consistent with the action potential-depressing properties of the agents [16]. XApEn computed from strongly lowpass-filtered versions of the data segments (cutoff frequency of 30 Hz as opposed to 200 Hz) yielded qualitatively comparable, but numerically inferior results (Additional file 1, Figure S1A), suggesting that signal changes in both low and high frequency ranges contribute to the anesthetic-induced decrease of XApEn.

## Discussion

XApEn calculated from the LFP of closely spaced intracortical sites showed significant changes with anesthetic concentrations. This finding demonstrates that volatile anesthetics coerce small cortical sub-networks, here represented by rat barrel cortex, into uniform, synchronized activity patterns.

Barrel cortex forms a large part of rodent somatosensory cortex, characterized by a one-to-one correspondence between the sensory organs (follicles at the base of the large facial whiskers) and cytoarchitectonically segregated structures in layer 4 termed 'barrels' [35-37]. This columnar, somatotopic organization results in an orderly bottom-up spread of sensory-evoked activity which, in the initial stage of processing, is spatially restricted to the discrete termination zones of the major thalamic afferents [38-40]. Yet, barrel cortex is also characterized by a large degree of synaptic divergence and interconnectivity, characteristic of neocortex in general [41]. Axons of pyramidal neurons may span several barrels, especially in layers 2/3 [40,42], and other cortical areas including contralateral barrel cortex form reciprocal, spatially dispersed connections [37,43]. Probably owing to this high degree of cortical interconnectivity, the permanent, ongoing activity therein, unrelated to sensory input, has a spatiotemporal profile which is largely independent of the barrel architecture [44,45]. In the present study, we recorded and analyzed this activity. Signal characteristics showed a dramatic and fundamental change during administration of volatile anesthetics.

The decrease of XApEn with all three volatile anesthetics suggests that these agents transform the diversity of synaptic inputs impinging on closely spaced pyramidal cells into a more uniform, synchronous pattern. These patterns may arise from an enhancement of GABAergic currents and a weakening of glutamatergic currents [46], which bias synaptic communication towards inhibition. Specifically, GABAergic interneurons likely gain in influence on cortical activity patterns by entraining local networks to common rhythms. Both the relative insensitivity of some interneuron classes to GABAergic inhibition [47] and the finding that inhibitory inputs in neighboring pyramidal neurons are more synchronous than excitatory inputs [20] are consistent with this idea. Furthermore, input via long-range connections from other cortical areas is impaired under anesthesia [9,48,49]. Experimental findings on figure-ground separation in monkey visual cortex could also be interpreted along the lines of a functional disconnection of cortical areas with anesthetics [50]. Therefore, it seems likely that the long-range excitatory synaptic inputs emanating from various cortical areas have an overall desynchronizing influence on local network activity in barrel cortex, and, by extension, that the impairment of this input by volatile anesthetics contributes to more local synchrony and thus a decrease of XApEn as observed.

Given that intracortical connections outnumber subcortical afferents [41] and that volatile anesthetics alter network activity in isolated cortical networks in vitro [16,51] we argue that the decline of XApEn was to a substantial part due to intracortical effects of the anesthetics. In addition, decreases in XApEn probably also reflect the anesthetic-induced transformation of activity in subcortical areas projecting to cortex. In particular thalamus, with its intricate reciprocal connections to cortex [36,37], must be considered [52]. Shown to be sensitive to volatile anesthetics and prone to bursting behavior, it may imprint its activity patterns on cortex [53-55]. Other likely candidates include the basal forebrain, which modulates cortical activity via cholinergic, GABAergic and glutamatergic afferents [56-58] as well as hypothalamic sleep pathways [59].

We found that the three volatile anesthetics differed in their potency to alter cortical activity patterns as quantified by XApEn. While isoflurane and enflurane were indistinguishable, halothane had significantly weaker effects. This finding fits the profile of this anesthetic, which has previously been found to exert weaker effects than isoflurane on XApEn computed from interhemispheric EEG [10] and on spontaneous action potential activity in neocortex in vitro and in vivo [16,51]. In rat visual cortex, Imas et al. found an enhancement of event-related gamma oscillations at intermediate concentrations of halothane [60], a finding which underlines the particular characteristic of this anesthetic. A potential limitation of our results is the fact that body temperature of the animals was not controlled, possibly leading to hypothermia during anesthesia. To minimize the influence of this phenomenon, the animals were placed into a plastic housing which provided some thermal insulation, and therefore at least it seems unlikely that they experienced severe hypothermia.

It is surprising that isoflurane- and halothane-induced changes of XApEn computed from interhemispheric EEG and LFP match qualitatively so well (Hudetz et al. used a different set of filter frequencies and computational parameters, so that a quantitative comparison of values may not lead to valid results [10,11]). First, although the basal mechanisms underlying EEG and LFP signals are identical - synaptic population currents in pyramidal cells giving rise to extracellular potential gradients - the populations of cells sampled from are not. EEG electrodes record potentials from a much larger population than intracortical electrodes. Furthermore, EEG signals are dominated by the largest dipole-generating contributors, pyramidal cells of layer 5, which extend their dendrites to layer 1. Intracortical signals, by contrast, are sampled from a restricted spatial volume [24] and are thus lamina-dependent [25,26]. Second, cortical sites separated by less than 1-2 mm as in our experiments receive a great deal of common synaptic input [20,61], and consequently exhibit more synchronous activity than cortical networks in different hemispheres. Thus, the qualitatively similar depression of XApEn values reported by Hudetz et al. and here show that anesthetics unfold synchronizing effects on widely different spatial scales.

## Conclusions

Anesthetics affect intracortical connections as well as corticocortical connections and inputs from subcortical areas. A disconnection or suppression of communication between different cortical areas is hypothesized to be a key player in the process of unconsciousness [1,62]. Buzsáki [4] suggested that local computations register in large parts of the cortex through long range-connections, and that this local-global wiring is necessary for subjective experiences. Our results suggest that local intracortical communication suffers from the uniformity of signals induced by volatile anesthetics, in parallel to a suppression or disconnection of long-range interareal connections as evident in EEG studies. Thus, our results underline the usefulness of multisite electrophysiological recordings - be it LFP in experimental animals or EEG in humans - in combination with XApEn and related parameters [29,63] for the quantification of anesthetic effects. By the same token, such approaches may serve to probe specific hypotheses on the neurophysiological correlate of consciousness, particularly those postulating a precisely timed convergence of synaptic inputs in neocortex [64], and possibly also to detect pathological connectivity between brain areas.

## Methods

### In vivo surgery & recording

All procedures described were in accordance with the policy on the use of animals in neuroscience research of the Society for Neuroscience and approved by the Ethical Committee on Animal Care and Use of the Government of Baden Württemberg, Germany. The experimental procedures were identical to those described in Hentschke et al [16] and the field potential data analyzed here were won from three of the four animals in the same study. Briefly, male or female Sprague-Dawley rats aged 12-16 weeks were anesthetized with ketamine/xylazine (100 mg/kg and 15 mg/kg, respectively). A craniectomy over the right hemisphere was performed, the dura was removed and a steel ring (O.D. 5 mm) placed on the pial surface. 4-16 custom-made etched tungsten microelectrodes (impedance 1-5 MΩ, tip separation ~180 μm) arranged as single or double linear arrays (one animal, 1 × 4; two animals, 2 × 8) were implanted through the steel ring into the neocortical somatosensory representation of the mystacial vibrissae ('barrel cortex'). Stereotactical target coordinates were Bregma -3.2 mm and lateral 4 mm. The array was oriented at an angle of 30-40° relative to the cortical surface such that the medial electrode row touched the cortical surface first and was thus located in a deeper cortical lamina than the lateral electrode row; the latter was aimed at the border of layers 4 and 5 (depth of penetration for this row from the point of contact with the pia was ~1100 um). Proper location of the electrodes within barrel cortex was verified by mapping of neuronal responses elicited by manual deflection of individual vibrissae. The electrode array and a head post were then fixed with dental cement and via 7-9 stainless steel screws driven into the skull. Recordings commenced after a recovery period of 2-4 d. The animals were head-restrained, sitting in a plastic housing, and placed in a sealable plexiglass box into which vaporizers (Drägerwerke, Lübeck, Germany) driven by air pumps delivered the anesthetics. For any given concentration, animals were exposed for periods of 27-39 min to the anesthetic. After discontinuation of the anesthetic, the animals were exposed to air for 40-60 min. A maximum of one recording session was performed per day.

Voltage traces recorded from the electrodes were referenced to the steel ring (rat1 and rat2) or to one of the electrodes (rat3), amplified and passed through a bandpass filter (-3 dB passband 0.5-200 Hz), digitized at 20 kHz and stored with a multichannel extracellular recording system (MultiChannelSystems, Reutlingen, Germany). Deteriorated, low-impedance electrodes with a small signal amplitude were excluded from analysis. The number of useable electrodes were 3 (rat3), 6 (rat2) and 12 (rat1). Anesthetic concentration is expressed in volume-% or as MAC (minimum alveolar concentration) as given in [65]: isoflurane 1.46%, enflurane 2.21%, halothane 1.03%.

### Selection of data segments

Under control conditions, the field potential data contained episodes of oscillatory activity with a dominant peak at 8-9 Hz (theta component) and an additional (non-harmonic) peak at 13-16 Hz (beta component; an example is given in Figure 3). The nature of these oscillations, also called 'high voltage rhythmic spikes' (HVRS), is a matter of debate. They appear in resting animals, often in conjunction with low-amplitude whisker movements [66] (whisker 'twitching') and may reflect a specific kind of idling, responsive state of the whisker sensory system [67-69]. Others consider HVRS manifestations of absence epilepsies [70-72]. We observed that at subanesthetic to hypnotic concentrations (isoflurane, 0.3-0.75%; enflurane, 0.5-1.2%; halothane, 0.65-1.05%) characteristics of the oscillations changed in several ways. Most notably, they were less coherent across channels and lasted for shorter periods. Furthermore, the dominant frequency was between 13 and 16 Hz. At the highest concentrations, which were equal to or above MAC (isoflurane, 1.45%; enflurane, 2.4%; halothane, 1.6%) the oscillations subsided and low frequency components and/or burst suppression patterns dominated.

We decided to exclude data with HVRS from analysis due to the unresolved nature of this activity and because the computational load of our analysis restricted the amount of data that could be analyzed in reasonable time to about 10 seconds per recording. To this end, the data acquired under control conditions were divided into segments of 2048 points overlapping by 730 points. From these, the spectral power of the signals in the range 7-9 Hz was computed for all channels. Of the resulting segment-wise power values, the 75^th ^and 90^th ^percentile were determined for each channel. We rejected segments with a power greater than the 90^th ^percentile on any of the recorded channels and/or with a power larger than the 75^th ^percentile on 50% or more of all recorded channels. The procedure was repeated for recordings with anesthetics, but with power determined in the range 10-16 Hz. Finally, within each recording, groups of four consecutive (overlapping) intervals which satisfied the criteria above were combined to yield segments of 6002 points length, corresponding to ~3 s. Three of such segments per recording, picked randomly from the beginning, middle and end of each recording were subjected to the subsequent analysis.

### Data analysis: Pearson's correlation and cross-approximate entropy

Cross-approximate entropy (XApEn) was introduced by Pincus for the analysis of hormone secretion processes [73]. It is an extension of approximate entropy which has been widely used in the analysis of biosignals such as EEG, electrocardiogram and, recently, local field potentials [29,74-76]. XApEn quantifies the predictability of the appearance of common patterns in two time series, not heeding the order in which they appear. Higher values of XApEn indicate higher degrees of independence or dissimilarity of the signals. As pointed out by Hudetz et al [10], XApEn is sensitive to changes in signal independence over a wide frequency spectrum and does not require stationarity of signals; thus it is very well suited to the analysis of local field potentials. In a preprocessing step, the selected data sequences were low pass filtered by a MATLAB 6.5 (The MathWorks, Inc., Natick, MA, USA) double reverse butterworth filtering routine (-3dB frequency of 200 Hz), which allows filtering with zero phase shift, and subsequently downsampled to 500 Hz. Next, data from each channel were normalized by subtracting the mean, followed by a division by the standard deviation. XApEn was calculated according to Pincus et al. [73]:

$$XApEn(m,r,N)(x||y)=\Phi^{m}(r)(x||y)-\Phi^{m+1}(r)(x||y)$$

Φ*^m^*(*r*)(*x *|| *y*)is the average of $\ln(C_{i}^{m}(x||y))$. $C_{i}^{m}(x||y)$ is the number of times which a sequence of defined length m in signal x starting at data point i has a similar counterpart anywhere in signal y, divided by N-m+1 (the number of comparisons possible). Two sequences are defined as similar if none of their scalar component differences (*x_i _*- *y_i_*) exceeds tolerance r (see Figure 2 for a detailed illustration of the computations). For analysis of the presented data, length m was set to 1 and tolerance r was 20% of the standard deviation of the signal in the channel combination with the lower channel number, i.e. if the channel combination was [1,4], SD was calculated for the signal recorded from channel 1. These settings are in accordance with the settings recommended by Pincus [73]. XApEn calculation was performed with MATLAB 6.5.

In addition to XApEn, Pearson cross correlation coefficient defined as

$$r=\frac{\sum_{i=1}^{n}\left(x_{i}-\bar{x})(y_{i}-\bar{y}\right)}{\sqrt{\sum_{i=1}^{n}{\left(x_{i}-\bar{x}\right)}^{2}\sum_{i=1}^{n}{\left(y_{i}-\bar{y}\right)}^{2}}},$$

where $\bar{x}$ and $\bar{y}$ are the mean of the intervals *x *= [*x*_1_, ..., *x_n_*] and *y *= [*y*_1_, ..., *y_n_*], was calculated using LabView 6i (National Instruments, Austin, Tx, USA). R [77]http://www.r-project.org/ was used for generating Figure 2.

## Competing interests

The authors declare that they have no competing interests.

## Authors' contributions

BA and GS conceived of the study and participated in its coordination. HH and CS performed animal surgery and the experiments. CS wrote data conversion code. MK performed cross-approximate entropy and cross correlation analysis and together with HH prepared the figures and performed statistical analyses. GS, BA, EK and CS participated in the preparation of the manuscript and interpretation of data. HH and MK wrote the paper. All authors read and approved the final manuscript.

## Supplementary Material

Additional file 1 **Figure S1A depicts the dose response relationships of XApEn for the same data as used in Figure **5**, but filtered with an upper cutoff frequency of 30 Hz and resampled at 100 Hz**. Same conventions as for Figure 5 apply, including monoexponential fits represented by solid lines. Qualitatively, the same observations as with the original data set, containing frequencies up to 200 Hz, can be made: decline of XApEn with anesthetic concentration, significant difference between halothane on the one hand and enflurane and isoflurane on the other hand. However, the data are more variable among animals and show less decline with anesthetic frequency. The free parameter tau was [95% confidence intervals]: 2.37 [1.34 3.41] for isoflurane, 2.30 [1.50 3.10] for enflurane and 6.31 [4.67 7.94] for halothane. R^2 ^values were 0.52, 0.68 and 0.51 (same order of agents). Figure S1B shows dose response relations of cross correlation values (normalized to control) computed from the same data as used for Figure 5. While on average the cross correlation increased with anesthetic concentration, there was little dose dependence, and intra- and interindividual variability was extremely high (cf. Figure 3). Click here for file
